# An assessment of population structure in eight breeds of cattle using a whole genome SNP panel

**DOI:** 10.1186/1471-2156-9-37

**Published:** 2008-05-20

**Authors:** Stephanie D McKay, Robert D Schnabel, Brenda M Murdoch, Lakshmi K Matukumalli, Jan Aerts, Wouter Coppieters, Denny Crews, Emmanuel Dias Neto, Clare A Gill, Chuan Gao, Hideyuki Mannen, Zhiquan Wang, Curt P Van Tassell, John L Williams, Jeremy F Taylor, Stephen S Moore

**Affiliations:** 1 Department of Agricultural, Food and Nutritional Science, University of Alberta, Edmonton, AB, Canada; 2 Division of Animal Sciences, University of Missouri, Columbia, MO, USA; 3 Bovine Functional Genomics Laboratory, U.S. Department of Agriculture, Agricultural Research Service, Beltsville, MD, USA; 4 Bioinformatics and Computational Biology, George Mason University, Manassas, VA, USA; 5 Division of Genetics and Genomics, Roslin Institute (Edinburgh), Midlothian, Scotland, UK; 6 Department of Genetics, Faculty of Veterinary Medicine, University of Liege, 4000-Liege, Belgium; 7 Agriculture and Agri-Food Canada Research Centre, Lethbridge, Alberta, Canada; 8 Instituto de Psiquiatria, Faculdade de Medicina – Universidade de São Paulo, São Paulo, SP, Brazil; 9 Genoa Biotecnologia S/A, São Paulo, SP, Brazil; 10 Department of Animal Science, Texas A&M University, College Station, Texas, USA; 11 Laboratory of Animal Breeding and Genetics, Graduate School of Science and Technology, Kobe University, Japan; 12 Parco Tecnologico Padano, Via Einstein, Polo Universitario, Lodi, Italy

## Abstract

**Background:**

Analyses of population structure and breed diversity have provided insight into the origin and evolution of cattle. Previously, these studies have used a low density of microsatellite markers, however, with the large number of single nucleotide polymorphism markers that are now available, it is possible to perform genome wide population genetic analyses in cattle. In this study, we used a high-density panel of SNP markers to examine population structure and diversity among eight cattle breeds sampled from *Bos indicus *and *Bos taurus*.

**Results:**

Two thousand six hundred and forty one single nucleotide polymorphisms (SNPs) spanning all of the bovine autosomal genome were genotyped in Angus, Brahman, Charolais, Dutch Black and White Dairy, Holstein, Japanese Black, Limousin and Nelore cattle. Population structure was examined using the linkage model in the program STRUCTURE and Fst estimates were used to construct a neighbor-joining tree to represent the phylogenetic relationship among these breeds.

**Conclusion:**

The whole-genome SNP panel identified several levels of population substructure in the set of examined cattle breeds. The greatest level of genetic differentiation was detected between the *Bos taurus *and *Bos indicus *breeds. When the *Bos indicus *breeds were excluded from the analysis, genetic differences among beef versus dairy and European versus Asian breeds were detected among the *Bos taurus *breeds. Exploration of the number of SNP loci required to differentiate between breeds showed that for 100 SNP loci, individuals could only be correctly clustered into breeds 50% of the time, thus a large number of SNP markers are required to replace the 30 microsatellite markers that are currently commonly used in genetic diversity studies.

## Background

Population structure and diversity within and between breeds of cattle have been studied to learn more about the origin, history and evolution of cattle [1-3]. Diversity studies and subsequent investigations concerning domestication events of *Bos taurus *and *Bos indicus *cattle have included sequencing from the displacement loop of mitochondrial DNA (mtDNA)[1]. Bradley *et al*. [1] used mtDNA sequence variation in 90 extant bovines from Africa, Europe and India to identify patterns of genetic variation consistent with the demographics of the domestication process. When nuclear marker have been used to study diversity in cattle, they have principally entailed microsatellite markers [2]. MacHugh *et al*. [2] used 20 microsatellites to help clarify the genetic relationships between cattle populations from Africa, Europe and Asia and provided support for a separate origin of domestication for *Bos taurus *and *Bos indicus *cattle. Analysis of allelic variation has been used to characterize the genetic relationships between breeds [4-7]. Kumar *et al*. [4] used 20 microsatellite markers to estimate the extent of genetic differentiation among breeds of cattle from India, Europe and the Near East. Assuming two ancestral populations, the mean admixture coefficients ranged from 0.0 to 0.1 in Indian *Bos indicus *breeds, 0.9 to 1.0 in European *Bos taurus *breeds and from 0.1 to 0.9 in hybrid breeds from the Near East. This variation in admixture coefficients reflects the ancestral divergence between the *Bos taurus *and *Bos indicus *subspecies. Similarly, Wiener *et al*. [5] characterized the diversity within and between eight British breeds of cattle using 30 microsatellite markers and found that the majority of the allelic variation (87%) was found within breeds. In addition, the studied breeds of cattle did not cluster according to their current geographic location, suggesting that the genetic origin of breeds was from different geographical regions. In a study of the origin of Chirikof Island cattle, MacNeil *et al*. [6] also found that 86% of the genetic variation in 34 microsatellite loci was found within *Bos taurus *breeds while the remaining 14% of genetic variation was found between breeds. However, the indigenous Chirikof Island cattle were strongly differentiated from the European *Bos taurus *cattle suggesting that a comparison between Asian *Bos taurus *breeds might next be appropriate. On the other hand, no significant divergence appears to exist between geographically separated populations of Holstein cattle probably due to historic occurrences of gene flow between populations and selection for similar traits [8]. Up to now most studies have focused on a small set of microsatellite loci, typically the 30 suggested by the FAO [9]. The true extent of autosomal diversity among cattle breeds has yet to be extensively explored. Here, we examine population substructure and interbreed diversity among eight breeds of cattle using 2,641 autosomal genome-wide SNPs.

## Results and Discussion

**Figure 1:**
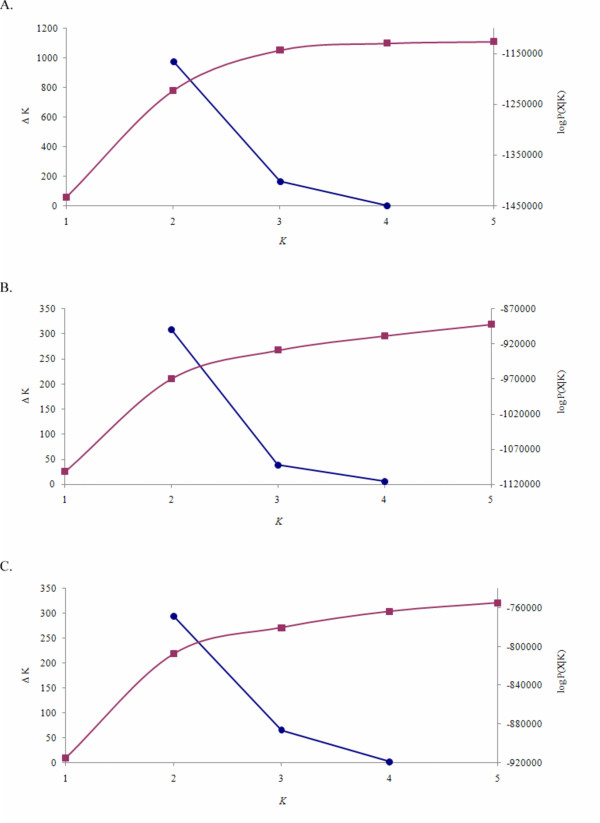
**(A-C). Δ*K *(-○-) and the mean log *P*(*X*|*K*) (-□-) based on the 5 replicate STRUCTURE runs indicate that *K *= 2 is optimal for each dataset**. The highest point on the blue line depicts the optimal *K *value. The red line depcits the mean log *P*(*X*|*K*) (-□-) for each K value. (A) All eight breeds included, (B) Only *Bos taurus *and (C) *Bos taurus *without Japanese Black.

**Figure 2:**
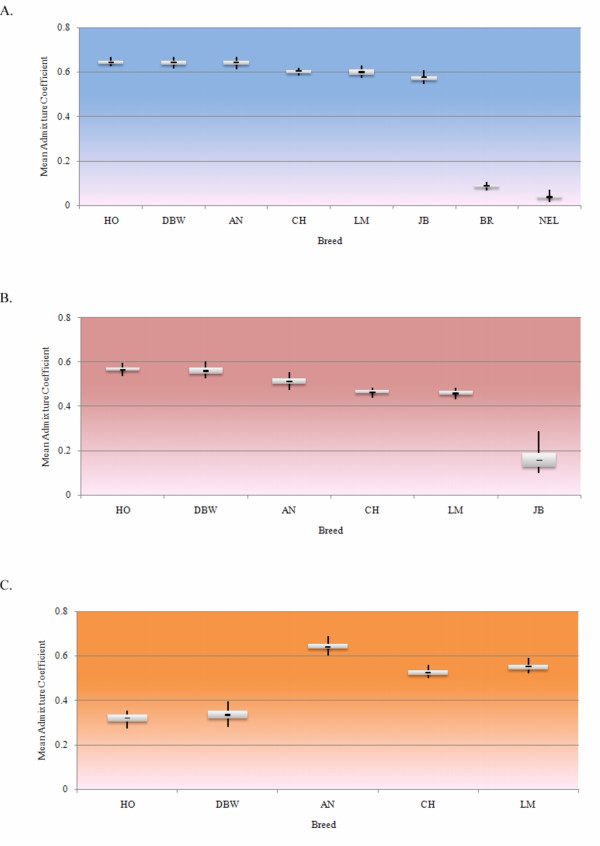
**(A-C). Box plot of mean individual admixture coefficients for the 5 replicate STRUCTURE runs using *K *= 2 for each dataset**. The boxes show the interquartile range of the mean individual admixture coefficients and contain 50% of the values. The black line shows the median value and the whiskers extend to the highest and lowest values. (A) All eight breeds included, (B) Only *Bos taurus *and (C) *Bos taurus *without Japanese Black. Breed abbreviations are defined in the text.

Preliminary analyses were performed using the STRUCTURE software. We first explored the appropriate number of iterations for the initial burn-in and estimation phases of the analysis. These preliminary analyses indicated that the probability of the number of ancestral populations (the *K *parameter from STRUCTURE) being greater than five was very small and therefore we restricted our analyses of all datasets to *K *≤ 5 to limit computation time (data not shown). Analyses were performed on three datasets which used the full complement of markers but varied according to breed representation. The first analysis included data for all eight breeds, the second dataset included only the six taurine breeds and the third analysis included all *Bos taurus *breeds excluding the Japanese Black. The number of ancestral populations (*K*) that were subsequently admixed to form these breeds was estimated using the method described by Evanno *et al*. [10] and was found to be no greater than two for each data set (Figure 1). The Δ*K *method of Evanno *et al*. [10] cannot be calculated at K = 1; however, the log-likelihood of the data, logP(X|*K*) in Figure 1, indicates that *K *= 1 can be excluded for all three of the analyzed datasets. The average estimated admixture coefficients (the *Q *parameters from STRUCTURE) of individuals from each breed are summarized in Figure 2 assuming two ancestral populations in each case.

The results presented in Figures 1a and 2a demonstrate that a considerable source of variation among cattle is the partitioning of breeds into the *Bos indicus *and *Bos taurus *subspecies. The variance between these groups (Table 1) accounted for 18.8% of the total variation (F_CT _= 0.19) which was significant (P ≤ 0.036). However, 71.06% of the genetic variation was found within populations. It should be noted that the SNP loci used in this study were detected in *Bos taurus *and their average minor allele frequency was much lower in *Bos indicus*. This ascertainment bias may have resulted in underestimated F_ST _values between breeds within both the *Bos taurus *and *Bos indicus *types, making them appear more similar than they really are and overestimating F_ST _values between the *Bos taurus *and *Bos indicus *breeds, making them appear more different than they really are. Despite this, the topography of the phylogeny and of breed composition from the STRUCTURE analyses should be correct, even if the distances between breeds are biased. Unfortunately, this supposition cannot be examined using our data, because the vast majority of the SNPs in the public domain and that were sampled for this study were discovered in *Bos taurus *cattle using procedures that guaranteed that the most common SNPs would be detected. The same problem exists for the studies that have historically employed microsatellite loci, since the sampled loci were cloned primarily from *Bos taurus *cattle and the loci selected for phylogenetic analysis were those possessing the most alleles when surveyed across populations. For example, *Bos taurus *and *Bos indicus *breeds have previously been clustered within subspecies using STRUCTURE in an analysis using 20 microsatellites. In a model with *K *= 2, Kumar *et al*. [4] found that the mean admixture coefficient of taurine breeds ranged from 0.9 to 1.0 while that for the indicine breeds ranged from 0 to 0.1. Our mean admixture coefficients ranged from 0.03 to 0.08 in *Bos indicus *and from 0.54 to 0.67 in *Bos taurus *breeds. While our findings are similar to those of Kumar *et al*. for the *Bos indicus *breeds, results for the *Bos taurus *breeds differ substantially. This may be due either to the difference in number of markers examined or, perhaps more likely, due to the different mutation rates between microsatellite and SNP loci. As the mutation rate for microsatellite loci is higher than for SNP loci [11], using microsatellite markers would most likely give higher estimates of divergence as measured by admixture.

**Table 1:** Analysis of Molecular Variance.

			Variance Components (%)				Fixation indices			
		
Data Set	# groups (*K*)	Among groups	Among populations within groups	Within populations	F_CT_	p value	F_SC_	p value	F_ST_	p value
All 8 breeds	2	18.79	10.15	71.06	0.19	0.036 ± 0.002	0.12	0.000 ± 0.000	0.29	0.000 ± 0.000
only *Bos taurus*	2	8.64	8.3	83.06	0.09	0.168 ± 0.003	0.09	0.000 ± 0.000	0.17	0.000 ± 0.000
*Bos taurus *without Japanese Black	2	4.65	5.59	89.76	0.05	0.101 ± 0.003	0.06	0.000 ± 0.000	0.10	0.000 ± 0.000

F_CT _is the correlation of random haplotypes within a group of populations, relative to that of random pairs of haplotypes drawn from the whole species. F_SC _is the correlation of the molecular diversity of random haplotypes within populations, relative to that of random pairs of haplotypes drawn from the region. F_ST _is the correlation of random haplotypes within populations, relative to that of random pairs of haplotypes drawn from the whole species.

To explore the population structure among the taurine breeds, a second STRUCTURE analysis was performed removing the two indicine breeds, and using data from the six taurine breeds (Figure 1b). This analysis identified Japanese Black cattle as being distinct from the cluster comprising the remaining five taurine breeds (Figure 2b). However, the partitioning was not strongly supported by the analysis of molecular variance (Fct = 0.09; P < 0.17; Table 1). The mean admixture coefficients for the European taurine breeds ranged from 0.43 to 0.60 while values for the Japanese Black ranged from 0.1 to 0.29. The upper and lower quartile range of the admixture coefficients for the individual Japanese Black animals were not as symmetric as found for the European taurine breeds (Figure 2b) and were skewed towards the European taurine breeds, suggesting a recent influence of European *Bos taurus *breeds within Japanese Black. Previously published reports describe the use of European breeds to upgrade Japanese Black cattle [12] which is supported by these data. Several domestication events have been suggested for cattle involving different strains of aurochs, including an independent taurine domestication event in Asia [12,13]. These results suggest that the Japanese Black breed is genetically distinct from the European taurine breeds and because the divergence greatly exceeds the variation between the beef and dairy breeds (Figure 2b), we believe that an independent Asian domestication event is more likely to explain the divergence than does selection or drift following domestication. The within breed variation in the admixture coefficient *Q *in Figure 2 also supports this contention. Provided the Japanese Black population does not represent a recent cross among divergent populations, the increased variation within this population is consistent with the hypothesis of a local Asian domestication event. Additional Asian derived cattle breeds will need to be tested to assess the weight of evidence for this hypothesis. However, our data are completely consistent with the origin of Japanese Black cattle being from an independent Asian domestication.

The third STRUCTURE analysis considered the remaining *Bos taurus *breeds after excluding the Japanese Black and resulted in a clustering of the meat and dairy breeds (Figures 1c, 2c). The mean admixture coefficients demonstrate considerably less variation within the Continental European breeds, which is consistent with the small effective population size that must have accompanied the introduction into North America of small samples of animals from these Continental breeds. The strong selection for milk production in the Holstein breed in conjunction with the extensive use of artificial insemination has reduced the genetic diversity within this breed and is apparent in these data. Surprisingly, therefore, the Dutch Black and White cattle had the greatest variation among all of the breeds studied suggesting that selection for milk production has been less intense in this breed than in Holsteins. Interestingly, 4.65% of the variation was found between the beef and dairy groups (F_CT _= 0.04) (Table 1) with a p value of 0.10 that was suggestive, but not significant. This variation suggests that artificial selection within cattle for alternate agricultural purposes has led to a genome wide divergence among the beef and dairy breeds. Additional analyses in which the genomic regions at which divergence between the types is greatest are overlaid with detected meat and milk QTL would be of considerable interest.

**Figure 3:**
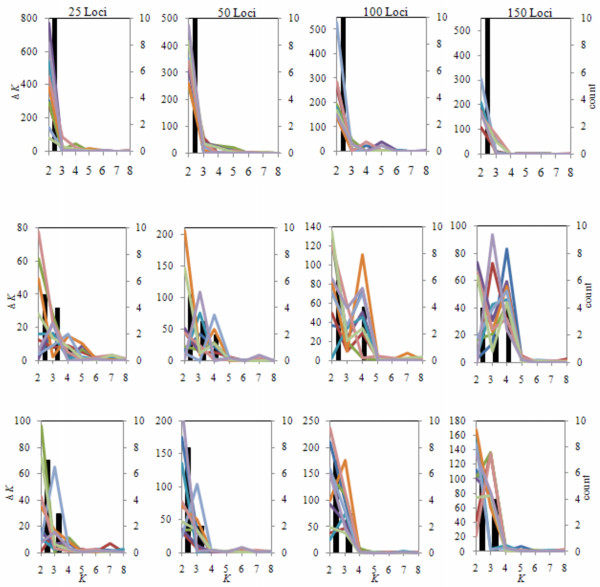
**(A-C). Estimation of the number of ancestral populations based on samples of 25, 50, 100 or 150 loci randomly sampled from the complete dataset**. In each panel, the solid bars represent the number of times each *K *was found to be optimal and the colored lines represent the Δ*K *values for each of the 10 replicate runs. (A) All eight breeds included, (B) All *Bos taurus *breeds and (C) *Bos taurus *without Japanese Black.

All of the STRUCTURE analyses using the three datasets supported the existence of two ancestral populations partitioning the breed types. Assuming that these represent the true number of ancestral populations, we sought to answer the question, how many loci would be required to precisely estimate the number of ancestral populations? We randomly sampled the dataset of 2,641 loci to produce 10 datasets with 25, 50, 100 or 150 loci and repeated each of the previous analyses. The results presented in Figure 3 show the results of each of the replicate analyses. At the subspecies level (Figure 3a), the correct number of ancestral populations was accurately inferred with as few as 25 loci. This is clearly due to the large divergence between the *Bos indicus *and *Bos taurus *subspecies which is demonstrated by the difference in the mean admixture coefficients in Figure 2a. The second analysis, which included only taurine breeds, demonstrates that using as many as 150 randomly chosen loci only yields the correct number of clusters in 40% of the instances (Figure 3b). This is most likely a result of the closer relationship between the taurine breeds (Figure 2b), and the presence of two levels of substratification among these breeds (Asian vs. beef vs. dairy). The third analysis, which excluded the Japanese Black breed, more frequently detected two ancestral populations (Figure 3c), which primarily detected the remaining beef vs. dairy strata in the data. Not surprisingly, it is evident from this set of analyses that the number of random SNP loci needed to accurately infer population structure is dependent on the divergence between populations. Earlier studies seeking to characterize the genetic diversity within and between breeds of cattle used 30 microsatellites [5]. Approximately three times the number of SNPs are needed compared to microsatellites [14], therefore, 150 SNPs should have been ample for inference of population structure. However, 150 SNPs still detected incorrect clustering among taurine breeds. Seldin *et al*. [15] reported similar results when trying to differentiate Northern and Southern European human populations using 400 randomly selected SNP loci and suggested the limited number of SNPs used as the potential problem. Clearly, future studies which seek to evaluate the relationships among closely related cattle breeds will require a larger number of SNP loci to accurately infer breed relationships.

**Figure 4:**
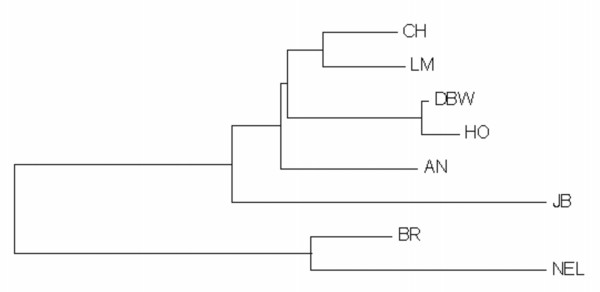
**Neighbor-Joining Tree based on pairwise Fst values calculated using 2,641 SNP loci showing phylogenetic relationships between cattle breeds**. Breed abbreviations are defined in the text.

Finally, we generated pairwise population Fst estimates using the complete dataset of 2,641 loci (Table 1) and used these to construct an unrooted Neighbor-Joining tree (Figure 4). This analysis is consistent with expectations, with the two French breeds clustering together, the Dutch Black and White and Holstein also clustered, with short branch lengths. The European group of Limousin, Charolais, Angus, Holstein and Dutch Black and White is separated from the Japanese Black, and the taurine breeds are separated from the indicine breeds. These estimates of genetic distance and the tree topology support the findings of the STRUCTURE analyses.

## Conclusion

The recent completion of a draft bovine genome sequence assembly has provided sufficient numbers of SNP loci to replace microsatellite loci and augment mtDNA sequences for population genetic analyses in cattle. We have shown that SNP loci can be used to identify population substructure among cattle breeds. However, we have demonstrated that a large number of SNP loci must be used to obtain an equivalent degree of precision in estimates of diversity compared with microsatellite loci, due to the lower information content of individual SNP loci. At issue is the importance of ascertainment of these loci to the phylogenies that are constructed. Because the majority of available SNPs were detected as the most common SNP within *Bos taurus *breeds, certain biases must exist within the analyses. However, the extent of these biases can only be quantified when these analyses are repeated using unbiased samples of loci, which to date, do not exist.

## Methods

### DNA Collection

DNA was collected from the following *Bos taurus *breeds: 70 Angus (USA), 20 Canadian Angus, 40 Charolais (Canada), 97 Dutch Black and White dairy cattle (Belgium), 48 Holstein (USA), 65 Japanese Black (Japan) and 43 Limousin (USA). Additionally, DNA was collected from two *Bos indicus *breeds: 40 Brahman (USA) and 97 Nelore (Brazil). Family structure and the number of individuals per family varied between breeds but the general family structure consisted of a grandparent, parent and three or more progeny. To determine the phase of alleles on the chromosomes using linkage information, we selected small families where members within the families were closely related but the families themselves were as unrelated as possible. This three generation family structure allowed for the efficient estimation of marker phase relationships in the progeny and also produced the most likely phase relationships in each of the parents and grandparents.

### Marker Selection and Genotyping

A detailed description of the SNP loci used in this study and of the genotyping methods was presented in McKay *et al*. [16]. Briefly, sequence information for SNPs (see Additional file 1) was obtained from public databases and SNPs were genotyped as a GoldenGate^® ^assay using an Illumina BeadStation 500 G [17]. Loci included in this study met the following criteria; minor allele frequency (MAF) ≥ 0.05 in Angus based on previous screens (data not shown) and concordant locus order between radiation hybrid (RH) maps [18] and genomic sequence location. The software GENOPROB V2.0 [19,20] was used to assess genotype score quality and to produce whole chromosome phased maternal and paternal haplotypes based on the pedigree and map locations of the loci.

### Population Structure Analysis

STRUCTURE and the linkage model of Falush *et al*. [21] were used to evaluate the extent of substructure among contemporary breeds of European and Asian *Bos taurus *and *Bos indicus *cattle. Exploratory STRUCTURE runs were used to determine the optimum number of iterations for the initial burn-in and estimation phases of the analysis to ensure that the Gibbs sampler had explored a sufficiently large sample space to provide reliable posterior probabilities. From these preliminary analyses, we determined that an initial burn-in of 10,000 iterations followed by 10,000 iterations for parameter estimation was sufficient to ensure convergence of parameter estimates (data not shown). We performed a series of analyses (runs) that were based on inclusion of differing combinations of cattle breeds in an attempt to determine the minimum number of ancestral populations that were admixed to best explain the genomic architecture of the current set of breeds. The first run used all of the animals from all 8 breeds. The second run used the 6 taurine breeds (Angus, Charolais, Limousin, Dutch Black and White Dairy, Holstein and Japanese Black) while the third run used the taurine breeds without the Japanese Black. To estimate the number of populations (the *K *parameter of STRUCTURE), each of these three data sets was analyzed allowing the value of *K *to vary from 1 to 5 and each run was repeated five times to produce a total of 75 STRUCTURE runs. Using the method of Evanno *et al*. [10] we calculated Δ*K *which is an *ad hoc *quantity related to the second order rate of change of the log probability (likelihood) of the data Pr(X|K) (equation 12 in [22]) with respect to the number of population clusters *K*.

Assuming the full dataset of 2,641 loci would yield the most accurate estimate of the true number of ancestral populations, we sought to determine the effect of the number of loci analyzed on inferences of *K*. Three new data sets each with 10 replicates were created by randomly sampling 25, 50, 100 or 150 loci from the 2,641 markers. Each replicate was analyzed using STRUCTURE as previously described except the admixture model was used rather than the linkage model as linkage among the sampled loci was assumed to be lost due to randomly sampling loci throughout the genome.

Finally, Fst values, population corrected average pairwise differences and analyses of molecular variance (AMOVA) were performed with the program ARLEQUIN [23] using the phased genotypes for each animal produced by GENOPROB V2.0. Significance levels for variance components and F statistics were estimated using 10,000 permutations. MEGA [24] was used to construct a Neighbor-Joining tree from the pairwise Fst values (Table 2).

**Table 2:** Pairwise Fst values based on 2,641 SNP loci.

	AN	BR	CH	DBW	HO	JB	LM
BR	0.271						
CH	0.090	0.273					
DBW	0.105	0.269	0.089				
HO	0.111	0.290	0.094	0.015			
JB	0.185	0.324	0.165	0.183	0.195		
LM	0.088	0.276	0.055	0.097	0.101	0.168	
NEL	0.320	0.112	0.335	0.316	0.350	0.373	0.336

Breed abbreviations are defined in the text.

## Authors' contributions

SDMcK conceived the study and participated in its design, data collection, analysis and manuscript preparation. RDS participated in study design, data analysis and manuscript preparation. BMM participated in study design, genotyping and data analysis. JA provided bioinformatics support. WC organized collection of Dutch Black and White samples, DC provided Charolais samples, EDN organized collection of Nelore samples, CAG provided bioinformatics support and organized collection of Brahman samples, CG provided bioinformatics support, HM organized the collection of Japanese Black samples, LKM provided bioinformatics support, ZW provided statistical support, CPVT made intellectual and bioinformatics contributions, JLW made intellectual contributions and helped in manuscript preparation, JFT participated in study design, provided Angus, Limousin and Holstein samples and helped draft the manuscript, SSM intellectual contributions. All authors read and approved the final manuscript.

## Supplementary Material

Additional file 1 **SNP information**. Map locations are shown in centimorgans for each SNP. Minor allele frequencies are shown for each SNP and each breed. Click here for file

## References

[B1] Bradley DG, MacHugh DE, Cunningham P, Loftus RT (1996). Mitochondrial diversity and the origins of African and European cattle. Proc Natl Acad Sci U S A.

[B2] MacHugh DE, Shriver MD, Loftus RT, Cunningham P, Bradley DG (1997). Microsatellite DNA variation and the evolution, domestication and phylogeography of taurine and zebu cattle (Bos taurus and Bos indicus). Genetics.

[B3] Troy CS, MacHugh DE, Bailey JF, Magee DA, Loftus RT, Cunningham P, Chamberlain AT, Sykes BC, Bradley DG (2001). Genetic evidence for Near-Eastern origins of European cattle. Nature.

[B4] Kumar P, Freeman AR, Loftus RT, Gaillard C, Fuller DQ, Bradley DG (2003). Admixture analysis of South Asian cattle. Heredity.

[B5] Wiener P, Burton D, Williams JL (2004). Breed relationships and definition in British cattle: a genetic analysis. Heredity.

[B6] Macneil MD, Cronin MA, Blackburn HD, Richards CM, Lockwood DR, Alexander LJ (2007). Genetic relationships between feral cattle from Chirikof Island, Alaska and other breeds. Anim Genet.

[B7] Negrini R, Nijman IJ, Milanesi E, Moazami-Goudarzi K, Williams JL, Erhardt G, Dunner S, Rodellar C, Valentini A, Bradley DG, Olsaker I, Kantanen J, Ajmone-Marsan P, Lenstra JA (2007). Differentiation of European cattle by AFLP fingerprinting. Anim Genet.

[B8] Zenger KR, Khatkar MS, Cavanagh JA, Hawken RJ, Raadsma HW (2007). Genome-wide genetic diversity of Holstein Friesian cattle reveals new insights into Australian and global population variability, including impact of selection. Anim Genet.

[B9] CaDBase. http://www.projects.roslin.ac.uk/cdiv/markers.html.

[B10] Evanno G, Regnaut S, Goudet J (2005). Detecting the number of clusters of individuals using the software STRUCTURE: a simulation study. Mol Ecol.

[B11] Vignal A, Milan D, SanCristobal M, Eggen A (2002). A review on SNP and other types of molecular markers and their use in animal genetics. Genet Sel Evol.

[B12] Mannen H, Tsuji S, Loftus RT, Bradley DG (1998). Mitochondrial DNA variation and evolution of Japanese black cattle (Bos taurus). Genetics.

[B13] Mannen H, Kohno M, Nagata Y, Tsuji S, Bradley DG, Yeo JS, Nyamsamba D, Zagdsuren Y, Yokohama M, Nomura K, Amano T (2004). Independent mitochondrial origin and historical genetic differentiation in North Eastern Asian cattle. Mol Phylogenet Evol.

[B14] Kruglyak L (1997). The use of a genetic map of biallelic markers in linkage studies. Nat Genet.

[B15] Seldin MF, Shigeta R, Villoslada P, Selmi C, Tuomilehto J, Silva G, Belmont JW, Klareskog L, Gregersen PK (2006). European population substructure: clustering of northern and southern populations. PLoS Genet.

[B16] McKay SD, Schnabel RD, Murdoch BM, Matukumalli LK, Aerts J, Coppieters W, Crews D, Dias Neto E, Gill CA, Gao C, Mannen H, Stothard P, Wang Z, Van Tassell CP, Williams JL, Taylor JF, Moore SS (2007). Whole genome linkage disequilibrium maps in cattle. BMC Genet.

[B17] Oliphant A, Barker DL, Stuelpnagel JR, Chee MS (2002). BeadArray technology: enabling an accurate, cost-effective approach to high-throughput genotyping. Biotechniques.

[B18] McKay SD, Schnabel RD, Murdoch BM, Aerts J, Gill CA, Gao C, Li C, Matukumalli LK, Stothard P, Wang Z, Van Tassell CP, Williams JL, Taylor JF, Moore SS (2007). Construction of bovine whole-genome radiation hybrid and linkage maps using high-throughput genotyping. Anim Genet.

[B19] Thallman RM, Bennett GL, Keele JW, Kappes SM (2001). Efficient computation of genotype probabilities for loci with many alleles: I. Allelic peeling. J Anim Sci.

[B20] Thallman RM, Bennett GL, Keele JW, Kappes SM (2001). Efficient computation of genotype probabilities for loci with many alleles: II. Iterative method for large, complex pedigrees. J Anim Sci.

[B21] Falush D, Stephens M, Pritchard JK (2003). Inference of population structure using multilocus genotype data: linked loci and correlated allele frequencies. Genetics.

[B22] Pritchard JK, Stephens M, Donnelly P (2000). Inference of population structure using multilocus genotype data. Genetics.

[B23] Schneider S, Roessli D, Excoffier L (2000). Arlequin: A software for population genetics data analysis.

[B24] Kumar S, Tamura K, Nei M (2004). MEGA3: Integrated software for Molecular Evolutionary Genetics Analysis and sequence alignment. Brief Bioinform.

